# Bilateral Femoral Neck Stress Fracture in an Obese Middle-Aged Female With Osteomalacia and Coxa-Vara Managed by Simultaneous Bilateral Total Hip Arthroplasty

**DOI:** 10.7759/cureus.11478

**Published:** 2020-11-13

**Authors:** Lavindra Tomar, Gaurav Govil, Pawan Dhawan

**Affiliations:** 1 Department of Orthopaedics, Max Super Specialty Hospital, Patparganj, New Delhi, IND; 2 Department of Orthopaedics, Max Super Speciality Hospital, Patparganj, New Delhi, IND

**Keywords:** femoral neck fractures, stress fractures, coxa vara, osteotomy, hip replacement

## Abstract

Femoral neck stress fractures in middle-aged individuals are usually associated with underlying metabolic bone disease. There is increased abnormal loading on femoral neck with associated coxa-vara and obesity in a female. Bilateral presentation of such fractures is rarely reported. Their management poses significant challenges. We present a case of bilateral femoral neck stress fracture in a 58-year-old obese housewife with compression type of the left hip fracture and tension type of the right hip fracture with non-union following an operated femoral neck stress fracture. She presented two months from the onset of symptoms and was treated with simultaneous bilateral total hip replacement for the management of her painful hips. Follow-up at 18 months revealed excellent outcome and return to independent painless daily routine activity. This report highlights the importance of early recognition of femoral neck stress fracture and challenges in the management of their late presentation. Total hip arthroplasty allows early mobilization with a favorable functional outcome.

## Introduction

Femoral neck stress fractures (FNSFs) account for 5% of all stress fractures [1,2]. Most of the reported FNSFs are unilateral [2-4]. These fractures are commonly reported in military recruits, long-distance runners, and older adults [2].

Bilateral FNSFs in middle-aged nonathletic individuals have been rarely reported [5]. Associated risk factors included abnormal anatomy, elderly females, seizures, renal osteodystrophy, long or short-term use of corticosteroid, amenorrhea, and osteomalacia [2-6]. Late presentation poses additional management challenges.

Compression type FNSFs are seen to occur inferior-medial on the femoral neck while tension type are superior-lateral [1,4,6]. Early diagnosis is a key to prevent displacement and collapse [2,6].

We present a case of bilateral femoral neck stress fracture in an obese female with osteomalacia and coxa-vara with an operated tension type stress fracture of the right hip with non-union and a compression type stress fracture of the left hip presenting with displacement, managed by simultaneous bilateral total hip arthroplasty (THA).

## Case presentation

A 58-year-old obese middle-aged housewife had a history of insidious pain in her right hip with difficulty in walking for the past three years. She went to a surgical centre for management in her native town and was diagnosed with a right femoral neck fracture. Further workup revealed osteomalacia and vitamin D deficiency. She underwent reduction and fixation with three cannulated cancellous screws for her initial management. Her medical management with calcium supplements and vitamin D was initiated. Restricted weight bearing with the use of crutches was allowed after six weeks and progressed to full weight-bearing within three months on the advice of her local practitioner. Though she was unable to ambulate without support since surgery, the ambulatory status further deteriorated in two months with a progressive left hip pain. She was no longer able to walk for daily routine activities. She presented to us with the relevant concern for treatment.

She complained of severe pain in both hips (left more than right) and marked difficulty in doing her routine daily activities including unrestricted utilization of toilet for the last two months. No history of trauma is reported. She was a postmenopausal obese female weighing 106 kilograms for her height (170 cm). Her body mass index (BMI) was 36.6 kg/m^2^. There was tenderness over bilateral hip joints (left more than right), external rotation deformity, and shortening of the left lower limb. Movement of the left hip joint was extremely painful and was not encouraged. There was pain at extremes of motion of the right hip joint. The patient was able to do straight leg raise on the right side but not on the left side. There was no distal neurovascular deficit in both lower limbs.

**Figure 1:**
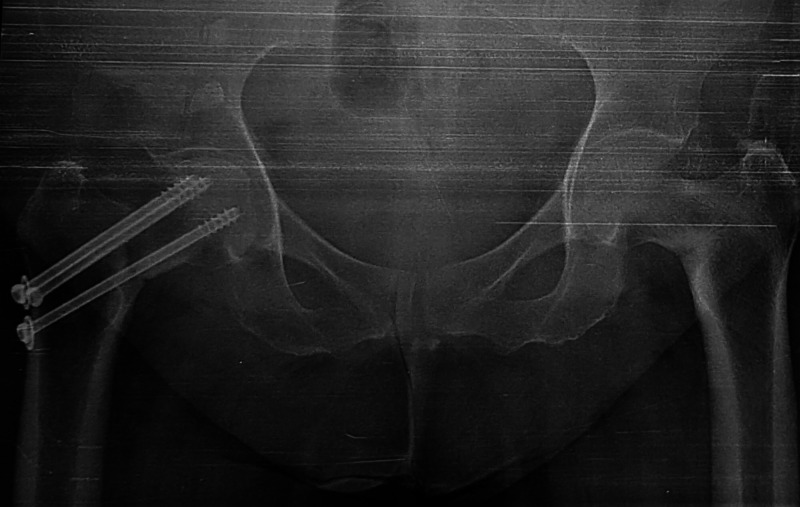
Anteroposterior both hips radiograph with right femoral neck fracture and initial screw fixation in situ with coxa-vara of left hip

Sequential radiographs were available and first radiograph showed fmoral neck fracture in the right lower limb with an initial in-situ fixation by three partially threaded screws (Figure 1).

**Figure 2:**
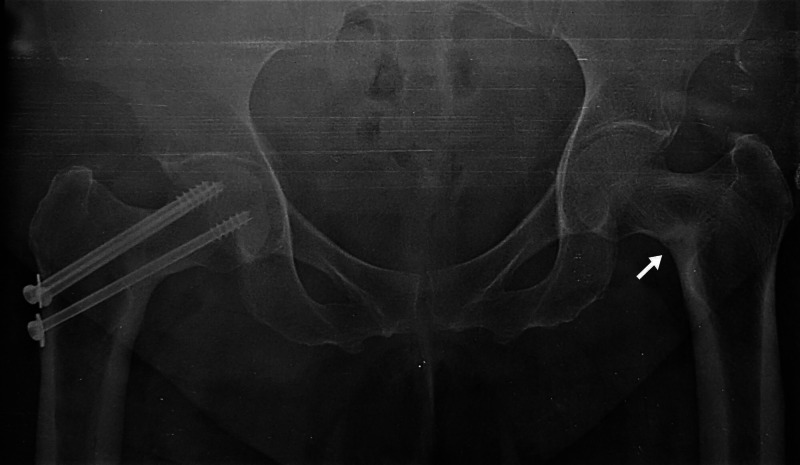
Anteroposterior both hip radiograph with right femoral neck fracture showing no avascular necrosis or collapse, bilateral varus angulation of femoral neck with sclerosis along inferomedial aspect of left neck femur (white arrow).

The second follow up radiograph at two years of right hip fracture fixation demonstrated no collapse or avascular necrosis of right hip fracture but with varus angulation of both hips with sclerosis along left femoral neck region concentrated along medial aspect with no fracture (Figure 2).

**Figure 3:**
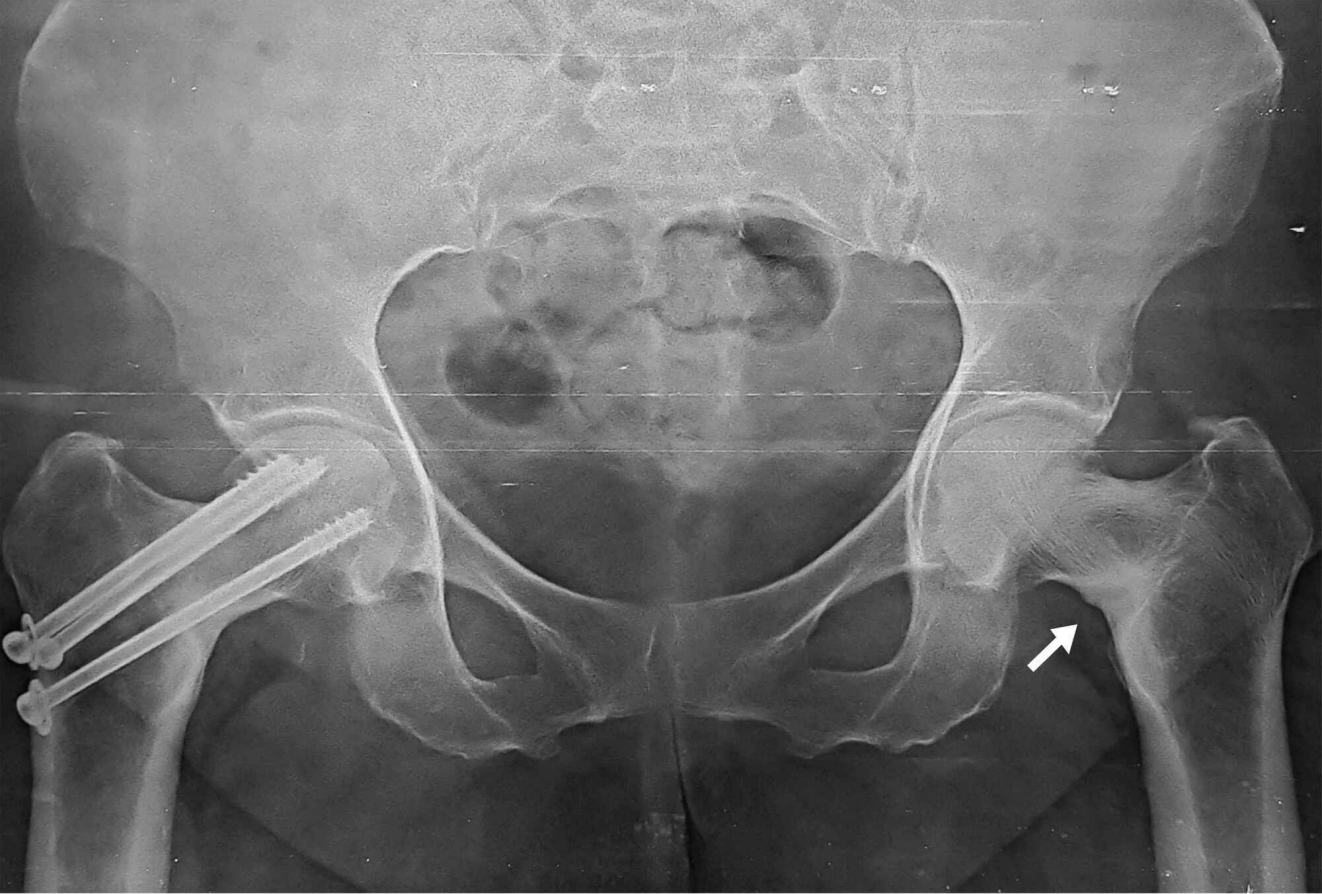
Anteroposterior both hips radiograph with increased sclerosis over bilateral inferomedial femoral neck with no fracture (white arrow).

The follow-up radiograph at three years showed persisting sclerosis along inferomedial aspect neck femur with a neck shaft angle of 115° on the right side and 117° on the left side (Figure 3).

**Figure 4:**
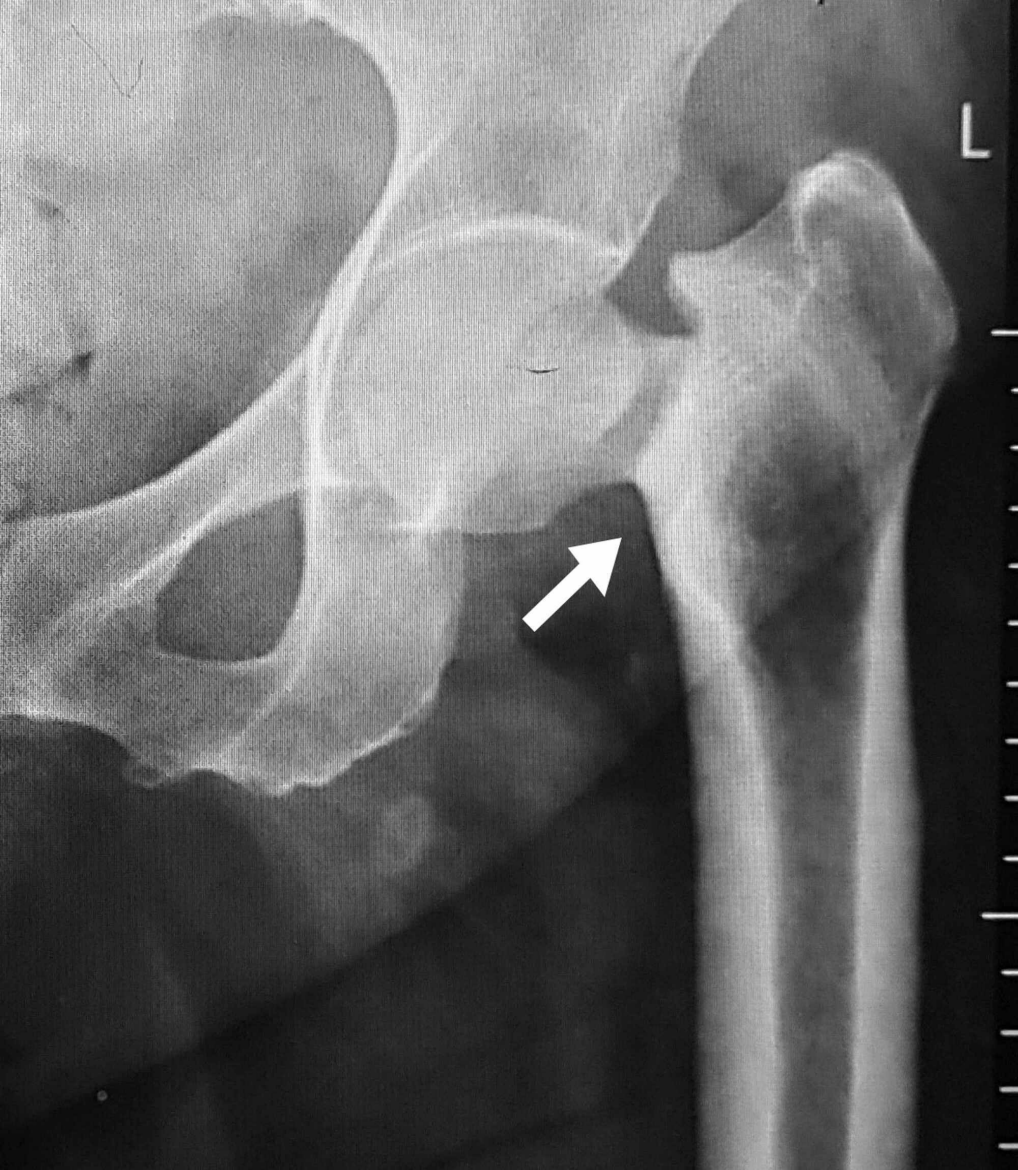
Anteroposterior left hip radiograph with displaced femoral neck fracture (white arrow).

**Figure 5:**
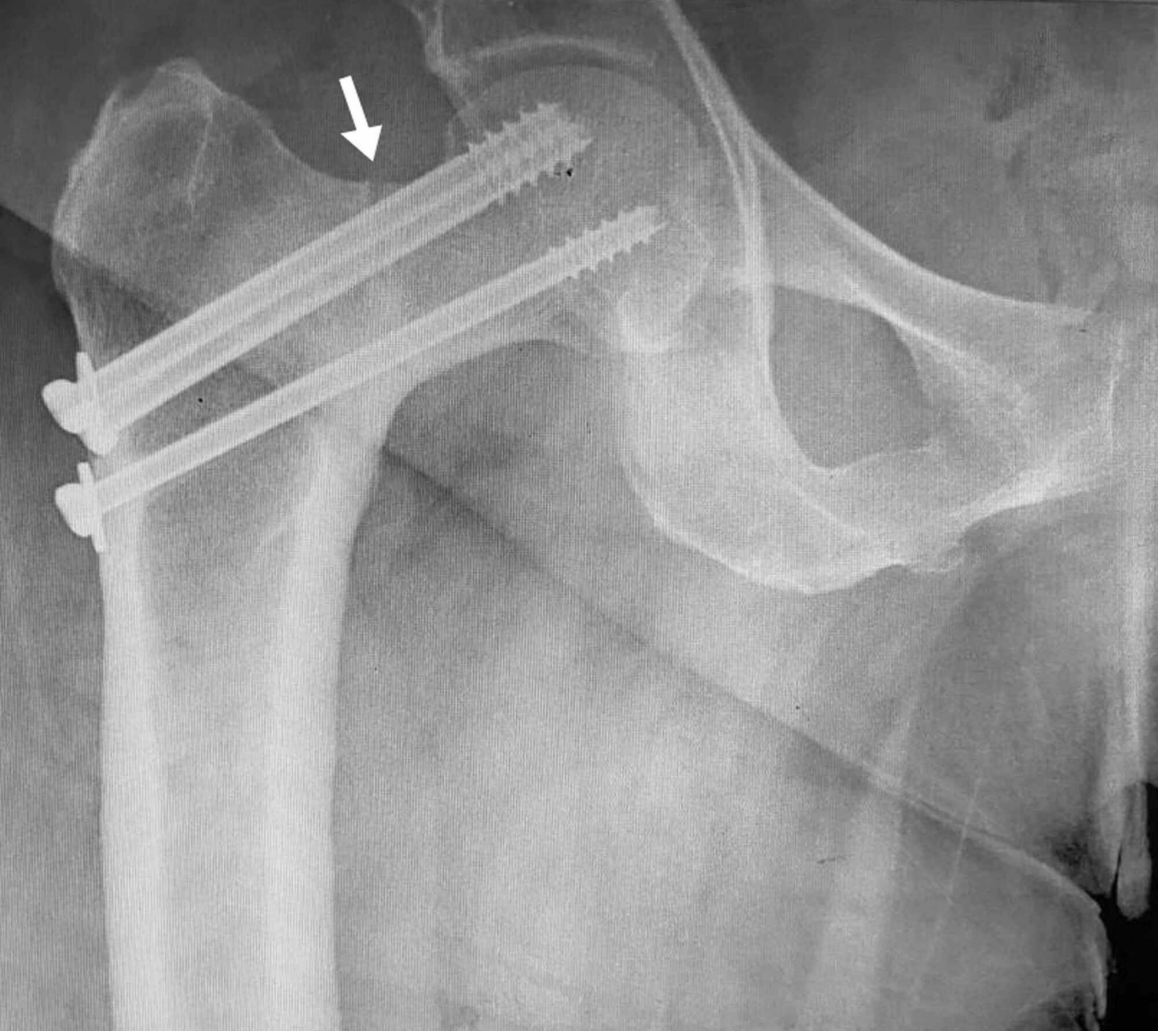
Anteroposterior right hip radiograph with radiolucent line along superolateral aspect likely tension type stress femoral neck fracture (white arrow).

The radiograph of hip joints at the time of presentation showed displaced femoral neck fracture left side (Figure 4) with right sided varus fixed neck femur fracture with screws in situ and radiolucent line along neck femur (Figure 5).

 

Whole spine radiographs revealed no abnormalities. The erythrocyte sedimentation rate (ESR) was measured 12 mm/hour and C-reactive protein (CRP) was qualitatively negative. Preoperative investigations included normal calcium, phosphorus, alkaline phosphatase, parathyroid hormone and 25-hydroxyvitamin D levels. Informed consent has been obtained from the patient to publish the data.

She underwent surgery in September 2018. The options of fixation and arthroplasty were discussed with the patient and planned for a bilateral THA. The goal was fracture management simultaneous with deformity correction (neck-shaft angle). The option of sequential, simultaneous or unilateral THA were discussed with the anaesthesia team and the patient. The patient was American Society of Anaesthesiologist grade 3 and considered for a simultaneous bilateral THA. Preoperative medications included intravenous antibiotic prophylaxis with injection cefuroxime and prophylactic subcutaneous enoxaparin. It was a single-stage bilateral total hip replacement. The surgery was done using combined spinal and epidural anaesthesia. Patient’s left hip operated first in a lateral position with a posterolateral approach. An uncemented total hip replacement was done with Depuy Johnson and Johnson implant (Pinnacle cup with poly-liner and Corail stem with large size 36 mm ceramic femoral head). The duration of surgery was 74 minutes. Right hip procedure followed in the same sitting with position change and re-draping. Upon removal of screws, fibrous union was detected with frank preoperative mobility at the fracture site conforming to the non-union of fracture. A hybrid THA was done with Depuy Johnson and Johnson implant (Pinnacle uncemented cup with poly-liner and cemented Corail stem with large size 36 mm ceramic femoral head). Duration of second surgery was 85 minutes. A preoperative haemoglobin level of 13.4 gm/dl fell to postoperative level of 10.4 gm/dl on day one postoperatively. Two units of packed red blood cells were transfused. Patient was ambulated with walker support from the second postoperative day. Postoperatively, she was given therapeutic doses of vitamin D and calcium supplements. She was discharged on the fifth day of the surgery. There were no immediate soft tissue or postoperative complications.

**Figure 6:**
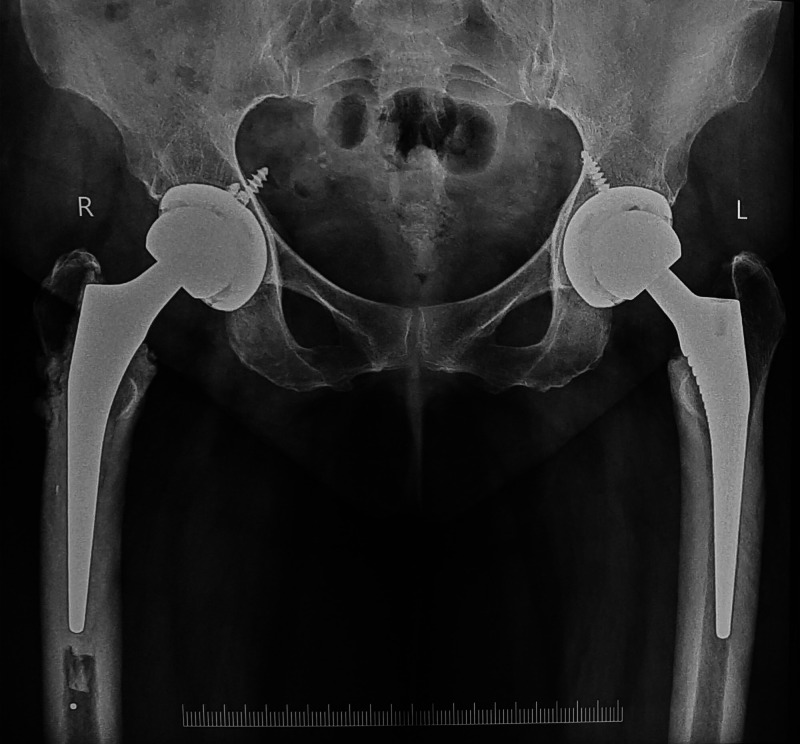
Anteroposterior both hips radiograph at two years follow up with bilateral total hip arthroplasty components in good alignment and no loosening

**Figure 7:**
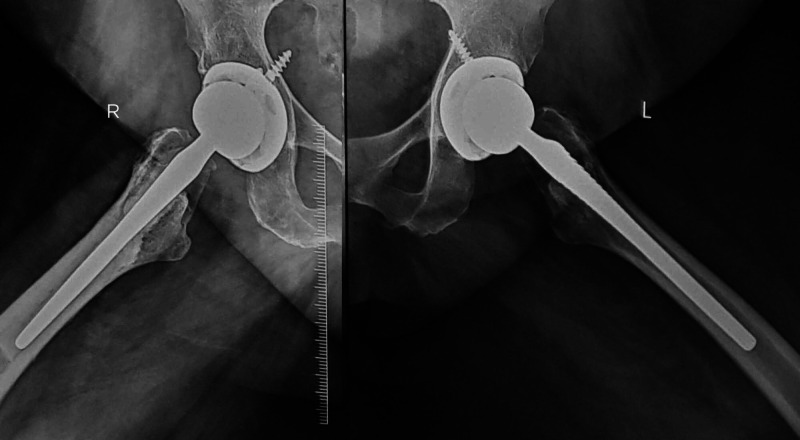
Lateral view both hips radiograph at two years follow up showed no component loosening or failure.

Patient at three months follow-up was clinically pain-free and was able to mobilize without support. She had no limb length discrepancy or gait abnormality. Patient at two years follow-up was pain-free and able to walk full weight-bearing without support and carry out her daily activities comfortably. Radiologically, well-positioned replacement components with no evidence of loosening or failure (Figures 6-7)

 

## Discussion

Stress fractures occur due to repetitive loading leading to mechanical failure of the bone. Such injuries occur either due to abnormal stresses on a normal bone (fatigue fractures) or normal stresses on an abnormal bone (insufficiency fractures) [4,6-8].

Fatigue fractures occur in the normal bone of a healthy individual as a result of excessive and repetitive loading. These fractures are common to athletes, dancers, and military personnel [4,7]. Insufficiency fractures occur in the weak bone under normal loading. There are several risk factors for these fractures such as osteoporosis, osteomalacia, long-term corticosteroid and anticonvulsant treatment, renal osteodystrophy, amenorrhea, fluoride treatment, and pelvic irradiation [4-7,9].

Factors causing bilateral femoral neck stress fracture should be carefully evaluated. One of them is osteomalacia, which is a common cause of insufficiency fractures, and the other one is coxa vara, a rare cause of fatigue fractures. Coxa vara exists when the femoral neck-shaft angle is less than 120 to 135 degrees. In the presence of abnormal hip anatomy, as in coxa vara, the deformity can produce a focal concentration of stresses in the femoral neck [4,7].

Vitamin D deficiency is a global health problem. Dietary deficiency-induced osteomalacia is one of the most common metabolic bone disorders [7,9]. In the presence of osteomalacia, the fracture callus usually does not form normally and healing occurs slowly as a result of delayed mineralization [3,9]. Prolonged immobilization after initiation of medical management in high doses may predispose to an episode of severe hypercalcemia [9]. Mobilization of the patient as quickly as possible after surgery will allow early resumption and initiation of medical treatment for osteomalacia [4].

A femoral neck stress fracture is classified as a high-risk fracture due to its location and potential for serious sequelae. Such high-risk stress fractures need to be diagnosed early and treated properly. Late presentations have severe complications of avascular necrosis or non-union with the onset of secondary hip arthritis [10].

Surgical treatment of late-presenting femoral neck stress fractures included either fracture fixation, valgus osteotomy or hip arthroplasty [5,7,11]. Fixation of femoral neck fractures is associated with a higher incidence of complications than any other fracture [11]. The reported incidence of avascular necrosis is 24% and non-union is 10% to 33% [1,2,5,11,12]. It is of paramount importance to evaluate the general condition of the patient, the fracture pattern, the implant selection amenable to fix that specific fracture, the surgical approaches, the anatomic reduction, and the technique of application of the implant to allow for early rehabilitation and mobilization.

The metabolic bone disease, deformity of the neck, morbid obesity, and coexistent poor healing in an operated hip in a middle-aged patient presents with significant management of challenges. We felt that an attempt to retain the patient’s femoral head in a femoral neck fracture with coexistent coxa-vara, osteopenia, and obesity in a 58-year old female with a corrective valgus osteotomy will predispose her to further prolonged period of limited mobilization and weight-bearing potential. Few reports on bilateral valgus osteotomy in FNSFs suggested an initial period of immobilization of two to three months duration before allowing weight-bearing [4,7]. Bilateral affection of hip compounds the weight-bearing potential after an osteotomy. Furthermore, the revision of valgus osteotomy to arthroplasty is even more demanding for the surgeon and patient alike. 

In a recent review of literature on bilateral FNSFs, there are very few reports of arthroplasty in a middle-aged patient treated by simultaneous THA for bilateral FNSFs [3,5]. There are no definitive suggestions regarding staged or simultaneous hip arthroplasty. Considerations for a cement-less or cemented arthroplasty and implant bearing selection are guided by age, pre-existing morbidities, available bone stock, and mobility [12]. High morbidity and mortality associated with increased systemic and local complications in a failed fracture fixation of neck femur make it imperative to minimize the failure of planned procedure for the patient’s age [12]. The ceramic on polyethylene bearing have superior survivorship in studies with long follow-up. A cement-less femoral component required a press-fit implantation with no evident per-operative loosening during left hip arthroplasty. The bone loss due to failed fixation and available poor bone stock during right hip arthroplasty was managed with a cemented femoral stem implantation. 

THA is considered a safe procedure with low mortality and complication rates [13]. Simultaneous hip arthroplasty procedures are more cost-effective, reducing overall anaesthetic time, length of stay, and rehabilitation [13]. In THA, there is a possibility of dislocation and component loosening [3,12]. However, a large femoral head of more than 32 mm increases jump distance and reduces rates of dislocation and the need for revision [14]. Although the decision making on the treatment is a matter of controversy, simultaneous bilateral total hip arthroplasty preserved mobility, and attained an early ambulatory status and good functional outcome.

## Conclusions

Emphasis is on early recognition of metabolic disorders and coexistent risk factors. There should be close monitoring by primary physicians to identify the pre-fracture stage for effective medical management of such entities. Late diagnosis is a matter of great concern as it may lead to significant surgical management challenges.

Simultaneous bilateral total hip arthroplasty for FNSFs is a reliable option in medically fit patients in a high-volume tertiary care arthroplasty unit with established postoperative protocols for rehabilitation and postoperative care for a good functional outcome.
